# Crystal structure of bis­(quinolin-1-ium) tetra­chlorido­ferrate(III) chloride

**DOI:** 10.1107/S2056989015024548

**Published:** 2015-12-31

**Authors:** Azzedine Boudjarda, Karim Bouchouit, Samiha Arroudj, Sofiane Bouacida, Hocine Merazig

**Affiliations:** a Faculté des Sciences Exactes et Informatique, Département de Chimie, Université de Jijel, 18000 Jijel, Algeria; b Laboratoire des Structures, Propriétés et Interactions InterAtomiques, Université de Khenchela, 40000 Khenchela, Algeria; c Unité de Recherche de Chimie de l’Environnement et Moléculaire Structurale, CHEMS, Université Constantine 1, 25000 , Algeria; d Département Sciences de la Matière, Université Oum El Bouaghi, Algeria

**Keywords:** crystal structure, hybrid compounds, tetra­chlorido­ferrate(III) anion, N—H⋯Cl hydrogen bonding

## Abstract

The asymmetric unit of the title hybrid compound, (C_9_H_8_N)[FeCl_4_]Cl, comprises a tetra­hedral tetra­chlorido­ferrate(III) anion, [FeCl_4_]^−^, a Cl^−^ anion and two quinolinium cations. There are N—H⋯Cl hydrogen-bonding inter­actions between the protonated N atoms of the quinolinium cations and the chloride anion, which together with π–π stacking between adjacent quinolinium rings [centroid-to-centroid distances between C_6_ and C_5_N rings in adjacent stacked quinolinium cations of 3.609 (2) and 3.802 (2) Å] serve to hold the structure together.

## Related literature   

For non-linear optical properties of hybrid compounds, see: Bouchouit *et al.* (2008 ▸, 2010 ▸, 2015 ▸); Jayalakshmi & Kumar (2006 ▸); Sankar *et al.* (2007 ▸). For similar structures containing the [FeCl_4_]^−^ anion, see: Khadri *et al.* (2013 ▸); Chen & Huang (2010 ▸); Prommon *et al.* (2012 ▸); Kruszynski *et al.* (2007 ▸).
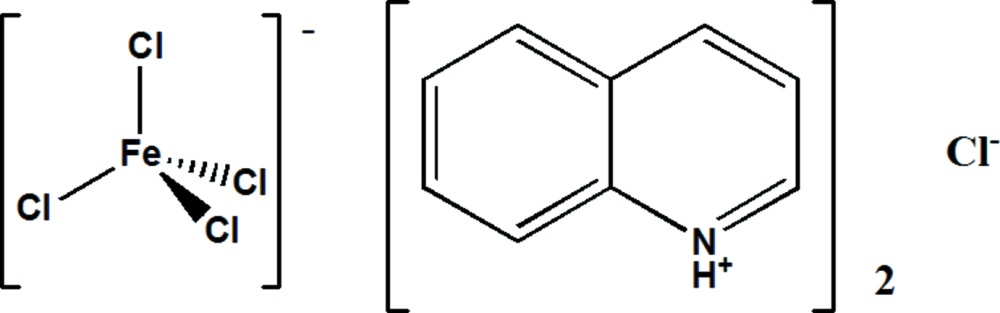


## Experimental   

### Crystal data  

(C_9_H_8_N)_2_[FeCl_4_]Cl
*M* *_r_* = 493.43
Triclinic, 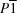

*a* = 8.424 (2) Å
*b* = 10.435 (3) Å
*c* = 13.022 (4) Å
α = 109.626 (18)°
β = 100.197 (19)°
γ = 90.893 (19)°
*V* = 1057.7 (5) Å^3^
*Z* = 2
Mo *K*α radiation
μ = 1.35 mm^−1^
*T* = 295 K
0.12 × 0.05 × 0.04 mm

### Data collection  

Bruker APEXII diffractometer
Absorption correction: multi-scan (*SADABS*; Sheldrick, 2002 ▸) *T*_min_ = 0.899, *T*_max_ = 0.922
9378 measured reflections
3738 independent reflections
2927 reflections with *I* > 2σ(*I*)
*R*_int_ = 0.043

### Refinement  

*R*[*F* ^2^ > 2σ(*F* ^2^)] = 0.033
*wR*(*F* ^2^) = 0.074
*S* = 1.01
3738 reflections
235 parameters
H-atom parameters constrained
Δρ_max_ = 0.29 e Å^−3^
Δρ_min_ = −0.31 e Å^−3^

### 

Data collection: *APEX2* (Bruker, 2011 ▸); cell refinement: *SAINT* (Bruker, 2011 ▸); data reduction: *SAINT*; program(s) used to solve structure: *SIR2002* (Burla *et al.*, 2005 ▸); program(s) used to refine structure: *SHELXL97* (Sheldrick, 2008 ▸); molecular graphics: *ORTEP-3 for Windows* (Farrugia, 2012 ▸) and *DIAMOND* (Brandenburg & Berndt, 2001 ▸); software used to prepare material for publication: *WinGX* (Farrugia, 2012 ▸).

## Supplementary Material

Crystal structure: contains datablock(s) I. DOI: 10.1107/S2056989015024548/cq2018sup1.cif

Structure factors: contains datablock(s) I. DOI: 10.1107/S2056989015024548/cq2018Isup2.hkl

**Fig. 1.**
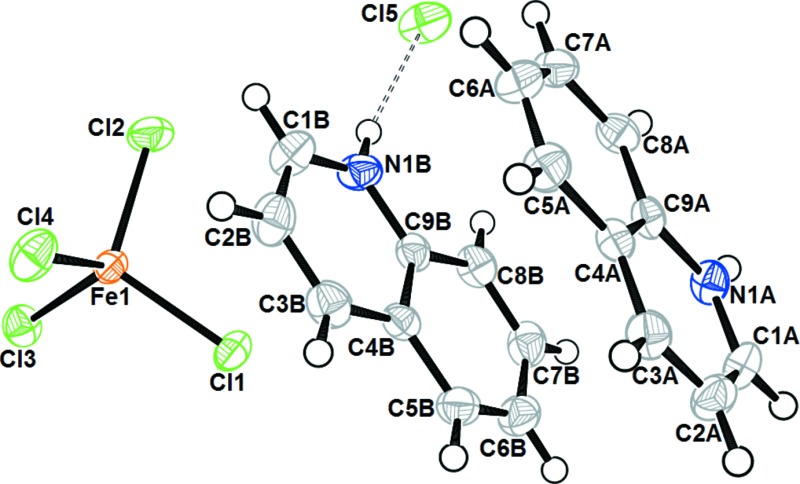
An ORTEP-3 (Farrugia, 2012) plot of the title compound, with the atom-numbering scheme. Displacement ellipsoids are drawn at the 50% probability level.

**Fig. 2.**
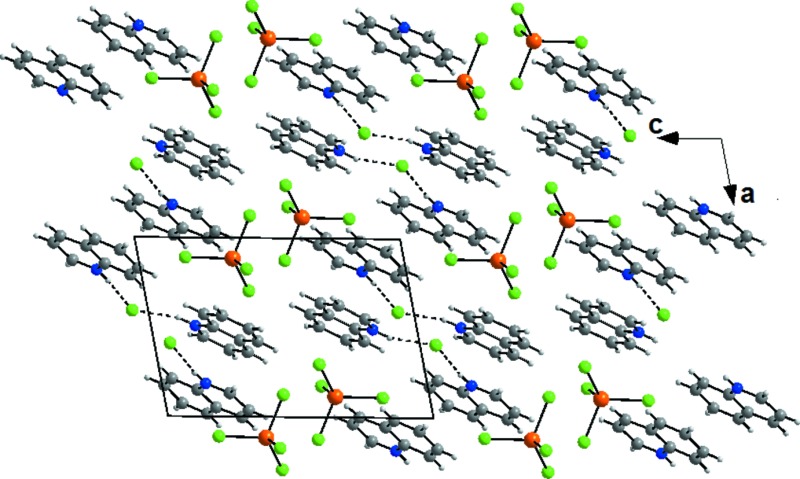
A packing diagram of the title compound, viewed along the b axis, showing the N—H···Cl hydrogen bonds as dashed lines.

ORTEP-3 . DOI: 10.1107/S2056989015024548/cq2018fig1.tif
An *ORTEP-3* (Farrugia, 2012) plot of the title compound, with the atom-numbering scheme. Displacement ellipsoids are drawn at the 50% probability level.

b . DOI: 10.1107/S2056989015024548/cq2018fig2.tif
A packing diagram of the title compound, viewed along the *b* axis, showing the N—H⋯Cl hydrogen bonds as dashed lines.

CCDC reference: 1443665

Additional supporting information:  crystallographic information; 3D view; checkCIF report

## Figures and Tables

**Table 1:** Hydrogen-bond geometry (Å, °)

*D*—H⋯*A*	*D*—H	H⋯*A*	*D*⋯*A*	*D*—H⋯*A*
N1*A*—H1*A*⋯Cl5^i^	0.86	2.16	3.014 (3)	174
N1*B*—H1*B*⋯Cl5	0.86	2.21	3.043 (3)	163

Symmetry code: (i) 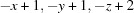.

## supplementary crystallographic information

### S1. Comment

Hybrid compounds are one of the important categories of materials. They have received much attention in research areas including nonlinear optics, second harmonic generation (SHG), third harmonic generation (THG) and optical switching [Bouchouit *et al.* (2008); Bouchouit, *et al.* (2010); Jayalakshmi *et al.* (2006); Sankar *et al.* (2007); Bouchouit *et al.* (2015)]. A considerable number of hybrid organic/inorganic compounds have been extensively studied for their promising properties. Crystals of many of these materials can be grown from aqueous solution (Khadri *et al.* (2013); Chen *et al.* (2010); Prommon *et al.* (2012); Kruszynski *et al.* (2007)]. In the present work, a mixture of water and acetonitrile is used as solvent for the reaction of quinoline with iron (III) chloride and leads to the generation of crystals of bis(quinolinium)tetrachloroferrate(III) chloride.

The asymmetric unit of the title hybrid compound consists of a tetrachloroferrate anion, (FeCl_4_)^−^, a chloride Cl^−^ anion and two quinolinium cations, (C_9_H_8_N)^+^ (Fig. 1). The iron atom lies at the centre of a regular tetrahedron and it is coordinated to four Cl atoms with Fe—Cl bond lengths in the range 2.1862 (10) to 2.2013 (10) Å. The lengths of the C–C and C—N bonds in the two independent quinolinium cations are comparable to the related distances found in the literature. The quinolium cations stack on top of each other, held together by π–π interactions. The centroid to centroid distances between C_6_ and C_5_N rings in adjacent stacked quinolinium cations are 3.609 (2) and 3.802 (2) Å.

The projection of the structure onto the *a*-*c* plane (Fig. 2) shows the N—H···Cl hydrogen bonding interactions between the N—H groups of the quinolium cations and the Cl^−^ anions which, together with the π–π interactions, serve to stabilize the structure.

### S2. Experimental

Quinoline, C_9_H_7_N, (0.2 mmol) and iron (III) chloride, FeCl_3_, (0.1 mmol) were dissolved in a mixture of water (10 ml) and acetonitrile (10 ml) at ambient temperature over a period of approximately 30 minutes. After this period, a brown precipitate appeared which was removed by filtration. The filtrate was then left at room temperature until brown crystals appeared.

### S3. Refinement

All non-H atoms were refined with anisotropic atomic displacement parameters. The remaining H atoms were localized on Fourier maps but introduced in calculated positions and treated as riding on their parent atom (C and N) with C—H = 0.93 Å and N—H = 0.86 Å with *U*_iso_(H) = 1.2 *U*_eq_

### Crystal data

**Table:**

(C_9_H_8_N)_2_[FeCl_4_]Cl	*Z* = 2
*M**_r_* = 493.43	*F*(000) = 498
Triclinic, *P*1	*D*_x_ = 1.549 Mg m^−^^3^
Hall symbol: -P 1	Mo *K*α radiation, λ = 0.71073 Å
*a* = 8.424 (2) Å	Cell parameters from 2434 reflections
*b* = 10.435 (3) Å	θ = 2.5–24.9°
*c* = 13.022 (4) Å	µ = 1.35 mm^−^^1^
α = 109.626 (18)°	*T* = 295 K
β = 100.197 (19)°	Prism, brown
γ = 90.893 (19)°	0.12 × 0.05 × 0.04 mm
*V* = 1057.7 (5) Å^3^	

### Data collection

**Table:**

Bruker APEXII diffractometer	2927 reflections with *I* > 2σ(*I*)
Graphite monochromator	*R*_int_ = 0.043
CCD rotation images, thin slices scans	θ_max_ = 25.1°, θ_min_ = 3.1°
Absorption correction: multi-scan (*SADABS*; Sheldrick, 2002)	*h* = −10→9
*T*_min_ = 0.899, *T*_max_ = 0.922	*k* = −12→12
9378 measured reflections	*l* = −15→15
3738 independent reflections	

### Refinement

**Table:**

Refinement on *F*^2^	Primary atom site location: structure-invariant direct methods
Least-squares matrix: full	Secondary atom site location: difference Fourier map
*R*[*F*^2^ > 2σ(*F*^2^)] = 0.033	Hydrogen site location: inferred from neighbouring sites
*wR*(*F*^2^) = 0.074	H-atom parameters constrained
*S* = 1.01	*w* = 1/[σ^2^(*F*_o_^2^) + (0.0268*P*)^2^ + 0.0315*P*] where *P* = (*F*_o_^2^ + 2*F*_c_^2^)/3
3738 reflections	(Δ/σ)_max_ = 0.001
235 parameters	Δρ_max_ = 0.29 e Å^−^^3^
0 restraints	Δρ_min_ = −0.31 e Å^−^^3^

### Special details

**Table:**

Geometry. All e.s.d.'s (except the e.s.d. in the dihedral angle between two l.s. planes) are estimated using the full covariance matrix. The cell e.s.d.'s are taken into account individually in the estimation of e.s.d.'s in distances, angles and torsion angles; correlations between e.s.d.'s in cell parameters are only used when they are defined by crystal symmetry. An approximate (isotropic) treatment of cell e.s.d.'s is used for estimating e.s.d.'s involving l.s. planes.
Refinement. Refinement of *F*^2^ against ALL reflections. The weighted *R*-factor *wR* and goodness of fit *S* are based on *F*^2^, conventional *R*-factors *R* are based on *F*, with *F* set to zero for negative *F*^2^. The threshold expression of *F*^2^ > σ(*F*^2^) is used only for calculating *R*-factors(gt) *etc*. and is not relevant to the choice of reflections for refinement. *R*-factors based on *F*^2^ are statistically about twice as large as those based on *F*, and *R*- factors based on ALL data will be even larger.

### Fractional atomic coordinates and isotropic or equivalent isotropic displacement parameters (Å^2^)

**Table:**

	*x*	*y*	*z*	*U*_iso_*/*U*_eq_	
Fe1	0.11617 (5)	−0.18619 (4)	0.64487 (3)	0.02192 (12)	
Cl4	0.29503 (9)	−0.33664 (7)	0.60671 (6)	0.03500 (19)	
Cl3	−0.12466 (8)	−0.28089 (7)	0.55023 (6)	0.02990 (18)	
Cl1	0.17477 (8)	−0.00943 (7)	0.59992 (6)	0.02792 (17)	
Cl5	0.40919 (9)	0.25683 (8)	1.05985 (6)	0.0361 (2)	
Cl2	0.11071 (11)	−0.11756 (8)	0.82228 (6)	0.0407 (2)	
N1A	0.8158 (3)	0.5396 (2)	0.83436 (18)	0.0227 (5)	
H1A	0.7565	0.5992	0.8689	0.027*	
N1B	0.4922 (3)	0.1123 (2)	0.83299 (18)	0.0263 (5)	
H1B	0.4486	0.1429	0.8906	0.032*	
C4A	0.9484 (3)	0.3350 (3)	0.8123 (2)	0.0209 (6)	
C3B	0.6376 (3)	0.0137 (3)	0.6543 (2)	0.0286 (7)	
H3B	0.689	−0.0202	0.5941	0.034*	
C3A	1.0018 (3)	0.3528 (3)	0.7215 (2)	0.0256 (6)	
H3A	1.0639	0.2886	0.6816	0.031*	
C8B	0.4286 (3)	0.3164 (3)	0.7925 (2)	0.0269 (6)	
H8B	0.3793	0.348	0.8539	0.032*	
C9A	0.8504 (3)	0.4318 (3)	0.8695 (2)	0.0214 (6)	
C2B	0.6258 (3)	−0.0606 (3)	0.7210 (2)	0.0317 (7)	
H2B	0.6671	−0.1457	0.7061	0.038*	
C6A	0.9269 (3)	0.2136 (3)	0.9376 (2)	0.0312 (7)	
H6A	0.9539	0.1416	0.9625	0.037*	
C7A	0.8267 (3)	0.3101 (3)	0.9917 (2)	0.0282 (7)	
H7A	0.7859	0.2996	1.0505	0.034*	
C7B	0.4349 (3)	0.3917 (3)	0.7256 (2)	0.0310 (7)	
H7B	0.3901	0.4754	0.7418	0.037*	
C4B	0.5727 (3)	0.1417 (3)	0.6753 (2)	0.0211 (6)	
C9B	0.4972 (3)	0.1909 (3)	0.7676 (2)	0.0217 (6)	
C5A	0.9848 (3)	0.2239 (3)	0.8496 (2)	0.0277 (6)	
H5A	1.0486	0.1579	0.8137	0.033*	
C1B	0.5514 (3)	−0.0082 (3)	0.8115 (2)	0.0319 (7)	
H1B1	0.5431	−0.0585	0.8576	0.038*	
C5B	0.5761 (3)	0.2235 (3)	0.6085 (2)	0.0274 (7)	
H5B	0.6258	0.1942	0.5471	0.033*	
C8A	0.7892 (3)	0.4185 (3)	0.9588 (2)	0.0244 (6)	
H8A	0.724	0.4826	0.9951	0.029*	
C1A	0.8687 (3)	0.5569 (3)	0.7502 (2)	0.0274 (7)	
H1A1	0.8423	0.6326	0.7302	0.033*	
C2A	0.9637 (3)	0.4630 (3)	0.6912 (2)	0.0282 (7)	
H2A	1.0007	0.4752	0.6317	0.034*	
C6B	0.5079 (3)	0.3445 (3)	0.6328 (2)	0.0309 (7)	
H6B	0.5097	0.3965	0.5873	0.037*	

### Atomic displacement parameters (Å^2^)

**Table:**

	*U*^11^	*U*^22^	*U*^33^	*U*^12^	*U*^13^	*U*^23^
Fe1	0.0269 (2)	0.0194 (2)	0.0219 (2)	0.00239 (16)	0.00657 (17)	0.00933 (17)
Cl4	0.0363 (4)	0.0294 (4)	0.0470 (5)	0.0126 (3)	0.0145 (4)	0.0193 (4)
Cl3	0.0271 (4)	0.0295 (4)	0.0319 (4)	−0.0008 (3)	0.0076 (3)	0.0082 (3)
Cl1	0.0317 (4)	0.0243 (4)	0.0322 (4)	0.0010 (3)	0.0065 (3)	0.0154 (3)
Cl5	0.0490 (5)	0.0359 (4)	0.0313 (4)	0.0214 (4)	0.0196 (4)	0.0149 (3)
Cl2	0.0691 (6)	0.0341 (4)	0.0230 (4)	0.0088 (4)	0.0136 (4)	0.0126 (3)
N1A	0.0248 (12)	0.0170 (12)	0.0259 (13)	0.0046 (9)	0.0097 (10)	0.0043 (10)
N1B	0.0307 (13)	0.0301 (14)	0.0209 (12)	0.0049 (11)	0.0092 (11)	0.0101 (11)
C4A	0.0192 (13)	0.0188 (14)	0.0216 (14)	0.0011 (11)	0.0015 (11)	0.0042 (12)
C3B	0.0278 (15)	0.0293 (17)	0.0266 (16)	0.0024 (13)	0.0097 (13)	0.0046 (13)
C3A	0.0248 (15)	0.0246 (16)	0.0260 (15)	0.0023 (12)	0.0104 (12)	0.0043 (13)
C8B	0.0255 (15)	0.0257 (16)	0.0272 (16)	0.0052 (12)	0.0086 (13)	0.0041 (13)
C9A	0.0180 (13)	0.0197 (15)	0.0228 (14)	−0.0033 (11)	0.0016 (12)	0.0041 (12)
C2B	0.0358 (17)	0.0210 (16)	0.0387 (18)	0.0083 (13)	0.0113 (15)	0.0085 (14)
C6A	0.0361 (17)	0.0306 (17)	0.0308 (17)	0.0038 (14)	0.0027 (14)	0.0175 (14)
C7A	0.0302 (16)	0.0335 (17)	0.0215 (15)	−0.0024 (13)	0.0030 (13)	0.0116 (13)
C7B	0.0296 (16)	0.0240 (16)	0.0372 (18)	0.0035 (13)	0.0004 (14)	0.0108 (14)
C4B	0.0190 (14)	0.0234 (15)	0.0186 (14)	−0.0006 (11)	0.0030 (11)	0.0048 (12)
C9B	0.0186 (14)	0.0222 (15)	0.0217 (14)	−0.0022 (11)	0.0011 (12)	0.0059 (12)
C5A	0.0278 (15)	0.0238 (16)	0.0315 (16)	0.0070 (12)	0.0041 (13)	0.0101 (13)
C1B	0.0357 (17)	0.0297 (17)	0.0359 (18)	0.0046 (14)	0.0097 (14)	0.0170 (14)
C5B	0.0268 (15)	0.0330 (17)	0.0223 (15)	−0.0012 (13)	0.0042 (13)	0.0099 (13)
C8A	0.0236 (14)	0.0273 (16)	0.0197 (14)	0.0036 (12)	0.0059 (12)	0.0039 (12)
C1A	0.0300 (16)	0.0217 (15)	0.0345 (17)	−0.0011 (12)	0.0081 (14)	0.0138 (13)
C2A	0.0302 (16)	0.0298 (17)	0.0296 (16)	−0.0002 (13)	0.0135 (13)	0.0127 (14)
C6B	0.0298 (16)	0.0327 (18)	0.0319 (17)	−0.0022 (13)	−0.0007 (14)	0.0169 (14)

### Geometric parameters (Å, º)

**Table:**

Fe1—Cl2	2.1862 (10)	C9A—C8A	1.399 (4)
Fe1—Cl1	2.1880 (9)	C2B—C1B	1.386 (4)
Fe1—Cl4	2.1901 (10)	C2B—H2B	0.93
Fe1—Cl3	2.2013 (10)	C6A—C5A	1.356 (4)
N1A—C1A	1.317 (3)	C6A—C7A	1.408 (4)
N1A—C9A	1.367 (3)	C6A—H6A	0.93
N1A—H1A	0.86	C7A—C8A	1.360 (4)
N1B—C1B	1.319 (4)	C7A—H7A	0.93
N1B—C9B	1.370 (3)	C7B—C6B	1.398 (4)
N1B—H1B	0.86	C7B—H7B	0.93
C4A—C3A	1.404 (4)	C4B—C9B	1.407 (4)
C4A—C9A	1.412 (4)	C4B—C5B	1.411 (4)
C4A—C5A	1.419 (4)	C5A—H5A	0.93
C3B—C2B	1.359 (4)	C1B—H1B1	0.93
C3B—C4B	1.410 (4)	C5B—C6B	1.358 (4)
C3B—H3B	0.93	C5B—H5B	0.93
C3A—C2A	1.362 (4)	C8A—H8A	0.93
C3A—H3A	0.93	C1A—C2A	1.388 (4)
C8B—C7B	1.361 (4)	C1A—H1A1	0.93
C8B—C9B	1.400 (4)	C2A—H2A	0.93
C8B—H8B	0.93	C6B—H6B	0.93
			
Cl2—Fe1—Cl1	108.97 (4)	C8A—C7A—C6A	120.6 (3)
Cl2—Fe1—Cl4	110.06 (4)	C8A—C7A—H7A	119.7
Cl1—Fe1—Cl4	110.70 (4)	C6A—C7A—H7A	119.7
Cl2—Fe1—Cl3	108.87 (4)	C8B—C7B—C6B	120.7 (3)
Cl1—Fe1—Cl3	109.03 (4)	C8B—C7B—H7B	119.6
Cl4—Fe1—Cl3	109.18 (4)	C6B—C7B—H7B	119.6
C1A—N1A—C9A	123.3 (2)	C9B—C4B—C3B	118.3 (2)
C1A—N1A—H1A	118.3	C9B—C4B—C5B	117.5 (2)
C9A—N1A—H1A	118.3	C3B—C4B—C5B	124.2 (2)
C1B—N1B—C9B	122.9 (2)	N1B—C9B—C8B	120.6 (2)
C1B—N1B—H1B	118.5	N1B—C9B—C4B	118.2 (2)
C9B—N1B—H1B	118.5	C8B—C9B—C4B	121.2 (2)
C3A—C4A—C9A	118.6 (2)	C6A—C5A—C4A	120.1 (3)
C3A—C4A—C5A	123.9 (3)	C6A—C5A—H5A	119.9
C9A—C4A—C5A	117.6 (2)	C4A—C5A—H5A	119.9
C2B—C3B—C4B	120.7 (3)	N1B—C1B—C2B	120.6 (3)
C2B—C3B—H3B	119.7	N1B—C1B—H1B1	119.7
C4B—C3B—H3B	119.7	C2B—C1B—H1B1	119.7
C2A—C3A—C4A	120.6 (3)	C6B—C5B—C4B	120.8 (3)
C2A—C3A—H3A	119.7	C6B—C5B—H5B	119.6
C4A—C3A—H3A	119.7	C4B—C5B—H5B	119.6
C7B—C8B—C9B	119.1 (3)	C7A—C8A—C9A	118.9 (3)
C7B—C8B—H8B	120.4	C7A—C8A—H8A	120.5
C9B—C8B—H8B	120.4	C9A—C8A—H8A	120.5
N1A—C9A—C8A	120.6 (2)	N1A—C1A—C2A	120.5 (3)
N1A—C9A—C4A	117.8 (2)	N1A—C1A—H1A1	119.7
C8A—C9A—C4A	121.6 (2)	C2A—C1A—H1A1	119.7
C3B—C2B—C1B	119.3 (3)	C3A—C2A—C1A	119.2 (3)
C3B—C2B—H2B	120.4	C3A—C2A—H2A	120.4
C1B—C2B—H2B	120.4	C1A—C2A—H2A	120.4
C5A—C6A—C7A	121.1 (3)	C5B—C6B—C7B	120.6 (3)
C5A—C6A—H6A	119.4	C5B—C6B—H6B	119.7
C7A—C6A—H6A	119.4	C7B—C6B—H6B	119.7
			
C9A—C4A—C3A—C2A	1.8 (4)	C5B—C4B—C9B—N1B	179.7 (2)
C5A—C4A—C3A—C2A	−179.2 (3)	C3B—C4B—C9B—C8B	−179.3 (2)
C1A—N1A—C9A—C8A	−179.4 (2)	C5B—C4B—C9B—C8B	−0.2 (4)
C1A—N1A—C9A—C4A	0.2 (4)	C7A—C6A—C5A—C4A	1.6 (4)
C3A—C4A—C9A—N1A	−1.4 (4)	C3A—C4A—C5A—C6A	−179.3 (3)
C5A—C4A—C9A—N1A	179.6 (2)	C9A—C4A—C5A—C6A	−0.3 (4)
C3A—C4A—C9A—C8A	178.2 (2)	C9B—N1B—C1B—C2B	1.3 (4)
C5A—C4A—C9A—C8A	−0.8 (4)	C3B—C2B—C1B—N1B	0.1 (4)
C4B—C3B—C2B—C1B	−1.1 (4)	C9B—C4B—C5B—C6B	−0.4 (4)
C5A—C6A—C7A—C8A	−1.8 (4)	C3B—C4B—C5B—C6B	178.6 (3)
C9B—C8B—C7B—C6B	0.4 (4)	C6A—C7A—C8A—C9A	0.7 (4)
C2B—C3B—C4B—C9B	0.7 (4)	N1A—C9A—C8A—C7A	−179.8 (2)
C2B—C3B—C4B—C5B	−178.3 (3)	C4A—C9A—C8A—C7A	0.6 (4)
C1B—N1B—C9B—C8B	178.2 (3)	C9A—N1A—C1A—C2A	0.6 (4)
C1B—N1B—C9B—C4B	−1.7 (4)	C4A—C3A—C2A—C1A	−1.0 (4)
C7B—C8B—C9B—N1B	−179.7 (2)	N1A—C1A—C2A—C3A	−0.1 (4)
C7B—C8B—C9B—C4B	0.2 (4)	C4B—C5B—C6B—C7B	1.0 (4)
C3B—C4B—C9B—N1B	0.6 (4)	C8B—C7B—C6B—C5B	−1.0 (4)

### Hydrogen-bond geometry (Å, º)

**Table:**

*D*—H···*A*	*D*—H	H···*A*	*D*···*A*	*D*—H···*A*
N1*A*—H1*A*···Cl5^i^	0.86	2.16	3.014 (3)	174
N1*B*—H1*B*···Cl5	0.86	2.21	3.043 (3)	163

Symmetry code: (i) −*x*+1, −*y*+1, −*z*+2.

## References

[bb1] Bouchouit, K., Bendeif, E. E., EL Ouazzani, H., Dahaoui, S., Lecomte, C., Benali-cherif, N. & Sahraoui, B. (2010). *Chem. Phys.* **375**, 1–7.

[bb2] Bouchouit, K., Bougharraf, H., Derkowska-Zielinska, B., Benali-cherif, N. & Sahraoui, B. (2015). *Opt. Mater.* **48**, 215–221.

[bb3] Bouchouit, K., Essaidi, Z., Abed, S., Migalska-Zalas, A., Derkowska, B., Benali-cherif, N., Mihaly, M., Meghea, A. & Sahraoui, B. (2008). *Chem. Phys. Lett.* **455**, 270–274.

[bb4] Brandenburg, K. & Berndt, M. (2001). *DIAMOND*. Crystal Impact GbR, Bonn, Germany.

[bb5] Bruker (2011). *APEX2* and *SAINT*. Bruker AXS Inc., Madison, Wisconsin, USA.

[bb6] Burla, M. C., Caliandro, R., Camalli, M., Carrozzini, B., Cascarano, G. L., De Caro, L., Giacovazzo, C., Polidori, G. & Spagna, R. (2005). *J. Appl. Cryst.* **38**, 381–388.

[bb7] Chen, L.-Z. & Huang, M.-N. (2010). *Acta Cryst.* E**66**, m377. 10.1107/S1600536810007889 PMC2983884 21580488

[bb8] Farrugia, L. J. (2012). *J. Appl. Cryst.* **45**, 849–854.

[bb9] Jayalakshmi, D. & Kumar, J. (2006). *Cryst. Res. Technol.* **41**, 37–40.

[bb10] Khadri, A., Bouchene, R., Bouacida, S., Merazig, H. & Roisnel, T. (2013). *Acta Cryst.* E**69**, m190. 10.1107/S160053681300603X PMC3629474 23633992

[bb11] Kruszynski, R., Wyrzykowski, D., Styczeń, E. & Chmurzyński, L. (2007). *Acta Cryst.* E**63**, m2279–m2280.

[bb12] Prommon, P., Promseenong, P. & Chainok, K. (2012). *Acta Cryst.* E**68**, m211–m212. 10.1107/S1600536812002486 PMC3274928 22346875

[bb13] Sankar, R., Raghavan, C. M. & Jayavel, R. (2007). *Cryst. Growth Des.* **7**, 501–505.

[bb14] Sheldrick, G. M. (2002). *SADABS*. Bruker AXS Inc., Madison, Wisconsin, USA.

[bb15] Sheldrick, G. M. (2008). *Acta Cryst.* A**64**, 112–122. 10.1107/S0108767307043930 18156677

