# Supplementary figures and images for: Crystal structure of bis­(quinolin-1-ium) tetra­chlorido­ferrate(III) chloride
Source: Acta Crystallogr E Crystallogr Commun. 2015 Dec 31;71(Pt 12):m273–4. doi: 10.1107/S2056989015024548 (PMC4719870; doi:10.1107/S2056989015024548)

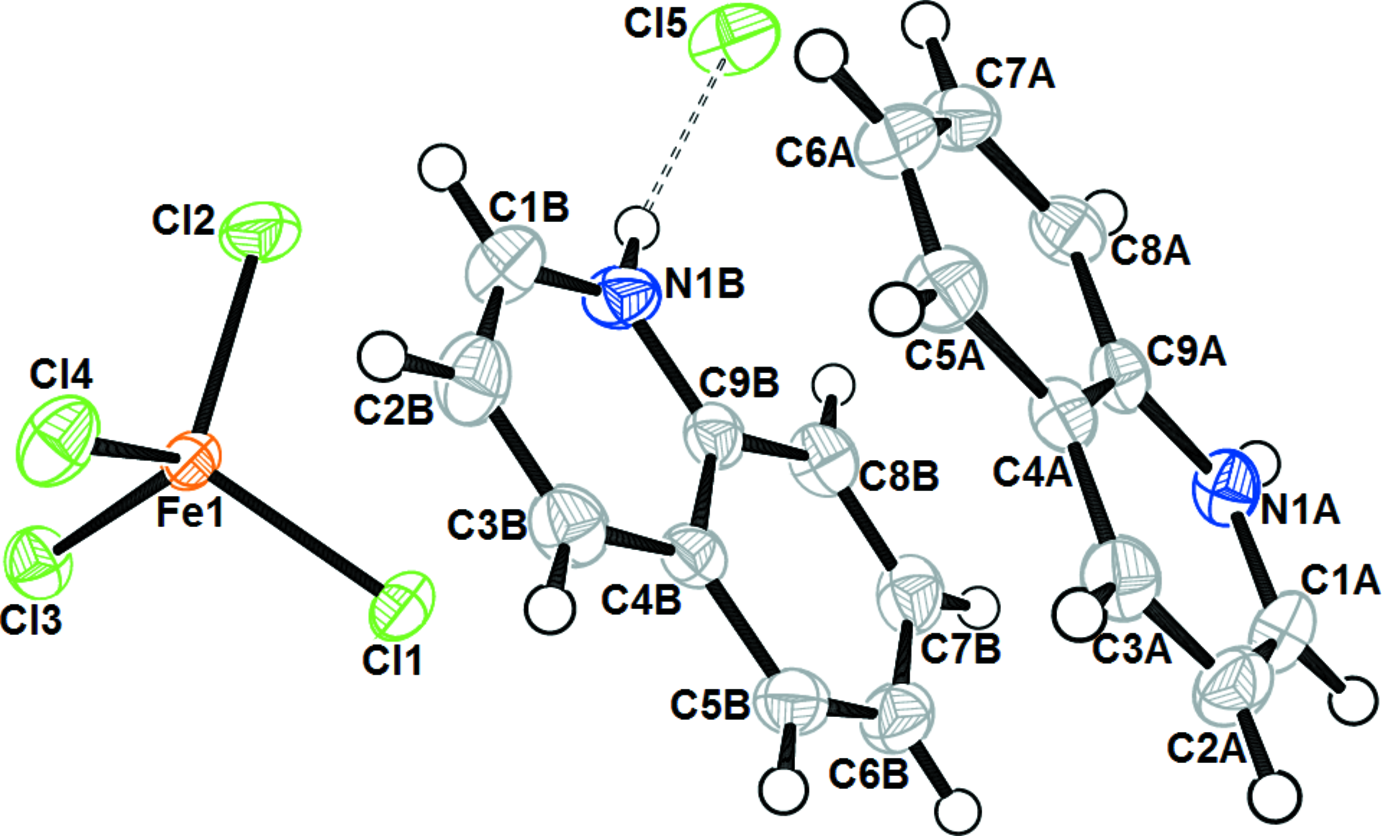

Supplement: Supplementary file 3 [file e-71-0m273-fig1.tif]

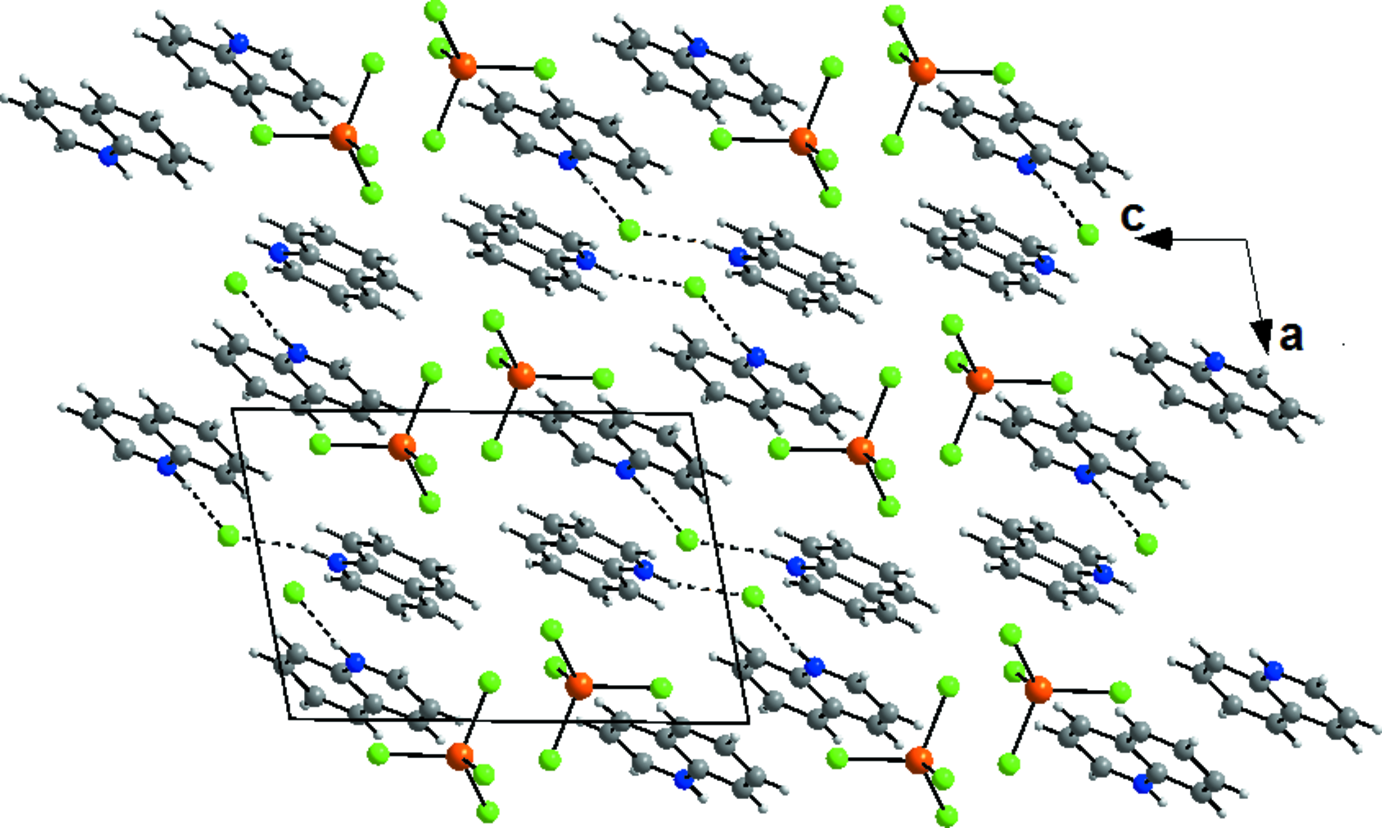

Supplement: Supplementary file 4 [file e-71-0m273-fig2.tif]
